# A healthy view? exploring the positive health perceptions of older adults with a lower socioeconomic status using photo-elicitation interviews

**DOI:** 10.1080/17482631.2021.1959496

**Published:** 2021-08-09

**Authors:** Feline Platzer, Nardi Steverink, Marieke Haan, Mathieu de Greef, Martine Goedendorp

**Affiliations:** a Department of Health Psychology, University Medical Center Groningen, Groningen, The Netherlands; b Faculty of Behavioural and Social Sciences, University of Groningen, Groningen, The Netherlands; c Department of Health Studies, Hanze University of Applied Science, Groningen, The Netherlands; d Department of Clinical Psychology and Experimental Psychopathology, University of Groningen, Groningen, The Netherlands

**Keywords:** Photo-elicitation interviews, lower socioeconomic status, qualitative research, positive health, self-management, older adults

## Abstract

**Purpose:** The health perceptions of older adults with a lower socioeconomic status still seems to be unsettled. To gain more insight in these perceptions, 19 older adults were interviewed with the use of a photo-elicitation method.

**Methods:** Participants reflected on ten photographs covering aspects of physical, social and mental health, and were also asked if and how they experience to have control over their health.

**Results:** The results showed that the perception of health depended on the background of the participant, was experience-oriented and was mostly focused on the negative aspects of physical and mental health. Social contacts were an important contributor to well-being, especially when physical health declined. Although most participants seem hardly aware of having influence on their own health, several participants showed automatic self-management abilities.

**Conclusion:** For participants who need more support to improve, or become more aware of their self-management abilities, interventions with an experience-oriented approach are needed.

## 1 Introduction

In a rapidly ageing population, maintaining a proper health status for as long as possible—also referred to as “ageing healthy and successfully”—is an overall goal for older adults, health professionals, and policy makers (World Health Organization (WHO), 2020). As a consequence, the terms “healthy ageing” and “successful ageing” are widely used in the literature. However, what “a proper health status” or “ageing healthy and successfully” exactly means for older adults themselves, is still unsettled.

In 1948 the World Health Organization (WHO) defined health as a “state of complete physical, mental, and social well-being, and not merely the absence of disease” in which health is more than a physical condition (World Health Organization (WHO), 2006). However, as Huber et al. (2011) convincingly argued, the 1948 definition of the WHO is not suitable anymore in an ageing society in which many people cope with chronic illnesses, because the WHO definition implies that a “complete” state of health should be achieved. Huber and colleagues proposed the new concept of “positive health” where health is seen as “the ability to adapt and self-manage in the face of physical, social and emotional challenges” (Huber et al., 2011, p. 2). Positive health, according to various groups of stakeholders, can be conceptualized by six domains, covering aspects of physical, social and mental health (Huber et al., 2016). In these terms, positive health does not focus on a “complete state” of health but rather on the achievement of, and satisfaction on, every domain for an individual. Although the study of Huber et al. (2016) resulted in six domains comprising the concept of positive health, there were still remarkable differences between stakeholders, especially between patients and health professionals, about the relative importance of the various domains. This indicates that the meaning and perception of health depends on the specific group to which respondents belong.

Research showed that especially for older adults with a lower socioeconomic status (also referred to as SES) the experience and meaning of health can be different than for health professionals. The association between lower SES and a higher risk of poor health is well documented (Präg et al., 2016). Socioeconomic status, which is related to educational level, income and occupation, is a robust factor that influences health from childhood on into older age, even across countries (World Health Organization (WHO), 2018). Adults with a lower SES are more likely to experience stressful life events, show more mental health problems, have a greater risk of diseases and of geriatric frailty, have less access to health care facilities, and have a greater risk of health-damaging behaviour, such as smoking (Loef & Walach, 2012; Szanton et al., 2010; Van Lenthe et al., 2004). As a result, individuals with a lower SES have a shorter overall life expectancy of, on average, seven years as compared to individuals with a higher SES. Moreover, individuals with a lower SES have a shorter life expectancy without any health problems of, on average, 18 years (Knoops & van den Brakel, 2010).

So far, only some studies investigated specifically the perception of health among older individuals with a lower SES. For example, in a quantitative study Barrett (2003) showed that the subjective experience of health in older adults with a lower SES is less positive than in older adults with a higher SES. The results of two other quantitative studies showed that older adults with a lower SES also tend to feel older than their chronological age, and expect their lifespan to be shorter than individuals with a higher SES (Baum & Boxley, 1983; Wardle & Steptoe, 2003). Barrett (2003) indicated that the experience of having older identities among older adults with a lower SES is partly due to being less optimistic about one’s health. Steverink et al. (2001) revealed a positive relationship between lower income and education level on the one hand, and the experience of ageing as a process of physical decline and social losses on the other hand.

It can be concluded that previous studies showed that older adults with a lower SES do perceive health in different ways than older adults with a higher SES. However, most studies used quantitative measurements, which reveal a restricted picture of the experiences and perceptions of health of this specific group because of the standardized measurements. For example, a study of Von Faber et al. (2001) showed that, when using standardized questionnaires with closed answer categories to assess physical, social, and psycho-cognitive functioning and well-being, only less than 10% (58/599) of the participants could be classified as “successfully aged”. However, when using qualitative (open) interviews with 27 participants, 22 older adults described themselves as “successfully aged”. Older adults seemed to use other criteria when expressing their own experiences than the predefined criteria in the quantitative study. An exception is the recent study of Flinterman et al. (2019) which used a qualitative focus group approach with concept mapping techniques to gain more insight into the perception of health in individuals with a lower SES. Results suggested that individuals with a lower SES, indeed, refer to other dimensions of health than other groups (e.g., patients, professionals, general population (Flinterman et al., 2019). However, the method of the latter study might still be debated, because concept mapping techniques require a certain amount of cognitive ability of the participants such as clustering and prioritizing (Flinterman et al., 2019). Many studies show the effect of a lower SES on the cognitive functioning of older adults (Kaplan et al., 2001; Lyu & Burr, 2016). It might, therefore, be questioned whether methods that require cognitive abilities such as reading, writing, and verbal expression, is adequate for older adults with a lower SES. The current study aims to gain more insight into the perception of health in older adults with a lower SES, using a visual method, namely photo-elicitation interviews (also referred to as PEI) (Mannay, 2013). The research question of this study is as follows: What are the positive health perceptions of older adults with a lower socioeconomic status, when older adults are interviewed with the use of photo-elicitation techniques?

## 2. Method

### 2.1 Ethical considerations

This study is part of a larger project (Community Wise) which focuses on the physical, social and mental health of low SES older adults by developing and testing a health-promotion intervention. For the entire project, the Ethics Review Board of the University Medical Center of Groningen examined the ethical considerations and granted exemption from further ethical review considering the minimal burden for the participants in 2016 (METc2016.498). By then, the regulations for non-medical research were less strict than they are now. During the study Dutch ethical and privacy laws and regulations changed, and we performed our research accordingly. We acted along the ethical rules of the Netherlands Organization of Health Research and Development. All data are anonymized according to the new privacy regulations. In addition, all participants gave their active written informed consent to participate in the study.

### 2.2 Design of the study

The current study aimed to gain more insight into the perceptions of positive health among older individuals with a lower SES by using PEI. Basically, PEI apply photographs taken by the researcher or participant in a research interview (Glaw et al., 2017; Harper, 2002; Mannay, 2013). The use of PEIs facilitates the communication between researcher and participant, and supports the associative thinking and reflection of the participant ((Glaw et al., 2017; Pain, 2012). Furthermore, the use of photos facilitates the memory and expression of ideas, and was, therefore, a suitable method to use in research with older adults with a lower SES (Collier & Collier, 1986; Copes et al., 2018; Pain, 2012). The photos that are used during the interviews are gathered by the researcher. One of the benefits of using photographs gathered by the researcher is the ability to trigger responses about concepts chosen by the researcher (Gong et al., 2012).

The photographs used in the current study focused on the concept of positive health and the biopsychosocial model of illness (Engel, 1977; Huber et al., 2011). The biopsychosocial model of illness from Engel (1977) defines health as more than only biomedical processes and focusses on the impact of psychological and social aspects on health. Consequently, the photographs used in the current study showed physical, social and mental aspects of health. We also focused on the concept of positive health defining health as “the ability to adapt and self-manage in the face of physical, social and emotional challenges of life” (Huber et al., 2011, p. 2). Therefore, we asked participants during the interview whether they believe to have any influence on health.

### 2.3 Gathering photographs & testing the photographs with representatives

The researcher searched on websites for available photos without portrait rights. Searching terms included e.g., *older adults and sports, older adults and social contacts, older adults and physical health*. The researcher asked for consent with the photographer when a suitable photograph had portrait rights. The first photograph selection was based on two criteria. First, the person displayed on the photo should be an older adult, and second, the photo needed to show a healthy and/or unhealthy situation in the domain of physical, social or mental health.

In cooperation with the research team, a first selection of 17 photographs was made. The researcher tested this selection of photographs during interviews with representatives of the target group. The researcher tested this selection of photographs during interviews with representatives of the target group. The representatives were people that were able to relate to the situation of the target group. Therefore, representatives belonged either to the target group (60 years or older and with a low SES) or were professionals working with the target group. Three representatives of the target group were above 60 years old (two with a low education level and one with a middle education level). The other three representatives worked as professionals with low SES older adults (one social worker, one health professional and one health care assistant). Four participants were female and two participants were male.

During these interviews, the representatives were asked to share reflections and associations on each photograph. This information was used to make adaptions in the selection of photographs. If a representative stated that the photograph was not appropriate to the situation of the target group, the feedback of the representative was discussed in the research team and another more suitable photograph was gathered online and tested with a different representative. For instance, a photograph were older adults were gardening did not fit the situation of most of the older adults in this study because these older adults lived in apartment buildings with no access to a garden. After testing 48 photographs with six representatives, a final set of 10 photographs was chosen to use in the interviews with the target group. We choose a restricted number of 10 photographs, to keep the duration of the interviews within a maximum of 60 minutes.

The final selection of photographs consisted of photographs that facilitated associations closest to the domains of physical, social and mental health. Eight out of the 10 final photographs fitted these three domains of positive health (*see* Table I). Due to the lower SES of the individuals, we choose to use two additional photographs relevant for the situation of the target group. One photograph showed the participant’s place of living (the village or apartment building), in order to investigate the associations of the living environment on the perception of health. This photograph was found online and was different for each participant depending on his or her place of living. The other photograph showed multiple banknotes with a total value of 70 euros, to evoke associations about participants’ financial situation in relation to their health perceptions.

**Table I.** Final selection of photographs used in the study (n = 10)

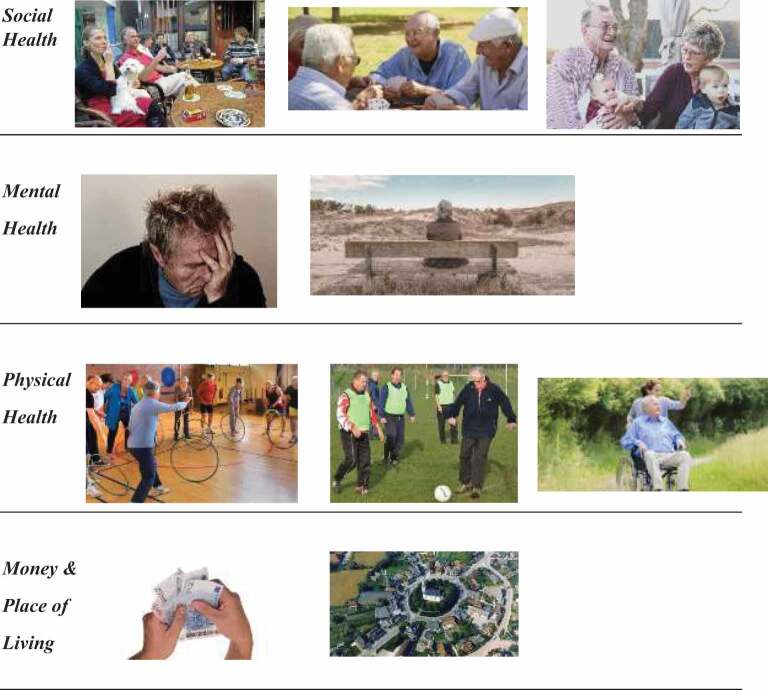

### 2.4 Interview questions

We strived to keep the interview schedule as open as possible to facilitate the associations of the participants. In general, we asked one question when showing the photograph:

*Can you tell me something about this photograph?*

The purpose of this question was to gain a first understanding of the associations of the participants. Depending on the participant’s degree of openness and answers, some additional questions were asked, such as:

*Is the situation on the photograph healthy?*

*Does the situation shown on the photograph have anything to do with health for you?*

*Are the people displayed on the photograph healthy?*

The financial status and education level of the participant were asked at the end of the interview. We choose to ask only at the end of the interview about financial status and educational level of the participant because of the sensitivity of the question. Doing so, we hoped that the established relationship between the interviewer and participant at the end of the interview would increase the chance that the participant would feel open to talk about their financial possibilities and education level, and consequently get less social desirable responses.

### 2.5 Pilot study: testing the photo-elicitation interview

The PEI with the 10 selected photographs was pre-tested with two individuals of the target group, in order to test the PEI method, the duration of the interview and the interview questions. The older adults who participated in the interviews were recruited at a retirement home. The interviews had a duration of 20 to 45 minutes. After the interview, participants were asked if the interview questions were clear and if they understood the PEI method. Participants showed no negative associations with the photographs and stated to have no additional feedback. Therefore, no changes were made in the design of the interview.

### 2.6 Participant selection

Participants needed to be above 60 years of age. We asked the age of the participant during the recruitment. Participants were recruited in neighbourhoods with inhabitants with a lower SES. Insight into the financial status of the participant was gained with the use of the photograph with banknotes. When discussing this photograph, participants could reflect on their own financial situation. If there was a need for more information about the financial situation of the participant, additional information about the income of the participants could be asked at the end of the interview. The level of education of the participant was asked at the end of the interview. The level of education of the participants were divided in lower, middle and higher education with the use of the classification criteria of the Statistics Netherlands (Statistics Netherlands, 2016). In this study, only older adults with a lower or middle level of education took part in the study.

Multiple methods were used to recruit participants. The intervention *“Community Wise”* aims to stimulate the positive health of older adults. Five participants were included with help of the recruitment for the intervention *“Community Wise”*. During this recruitment, older adults living in lower SES neighbourhoods in the northeast of the Netherlands were asked to participate in the intervention during a door-to-door interview method. When they indicated to have no interest in participating in the intervention, they were asked to take part in the current study. One older adult dropped-out of the first session of the *“Community Wise”* intervention and participated in the current study. Furthermore, organizations working with older adults with a lower SES, such as sports centres or foundations for older adults were asked to invite their members to take part in the study. Ten participants were recruited using the network of these organizations. Finally, we used snowball sampling, known as a method to include participants which are not easy accessible (Ghaljaie et al., 2017). After the interview with a participant, he/she was asked whether he/she knew other older adults in their network who might be interested to participate in the study. These participants asked in their network if someone wanted to take part in the study and exchanged the telephone number of the researcher in order to contact the researcher for an appointment. This method yielded another four participants, resulting in a total of 19 older adults participating in the study. None of the participants who signed up for the interview dropped out of the study.

### 2.7 Data collection and procedure

The 19 PEI were conducted in a five months period from November 2018 until March 2019. The researcher conducted 17 interviews; two were conducted by a research assistant. Both interviewers had experience with the target group and with semi-structured interviewing.

Saturation was achieved after coding 12 interviews, which meant that the researchers did not notice new information. However, after saturation occurred, we included more male participants in order to have more balanced insights of the perceptions of both male and female participants. With the aim to obtain more data in order to ensure that we gained a broad understanding of participants’ perceptions, also known as “meaning saturation”, more interviews were needed to properly understand the data (Hennink et al., 2017). The mean duration of the interviews was 35 minutes (SD = 11) ranging from 25 till 60 minutes.

The interview started with an introduction of the interviewer and the participant, after which participants were asked for consent to audio-record the interview. Before the start of the interview, participants received some brief information about the duration of the interview and participants were told that they were going to talk about ten photographs which displayed daily life situations. It was decided to debrief participants afterwards about the purpose and topic of the study, in order to avoid influencing participants’ associations about health at the start of the interview. Participants were told that the informed consent would be explained fully after the interview was finished. All participants gave their consent to use their data in the study. Although participants could stop the interview, no interview was prematurely truncated. Twelve interviews took place at participants’ homes, seven interviews took place in the canteen of the sports centre for older adults where the participants were recruited.

### 2.8 Data analysis

All interviews were transcribed verbatim, except for names and place of living of the participants which were substituted with functional codes to ensure the participants’ confidentiality. The transcripts were analysed to the standards of a thematic analysis, which is a suitable method for describing patterns in data and is used across a broad range of social research (Terry et al., 2017). A hybrid approach was used, which blends an inductive with a deductive approach to identify meaningful themes in the data (Boyatzis, 1998; Swain, 2018). Meaningful themes can be described as the capture of something important with regard to the research question (Swain, 2018; Terry et al., 2017).

During the data coding phase, small units of the transcripts were segmented by their meaning to codes. To ensure intersubjectivity, two researchers checked in different configurations the codes of the researcher and differences in coding were discussed in the whole research team. We coded the transcripts based on the biopsychosocial model of health (Engel, 1977) and focused on fragments about social, mental and physical health. After coding the first interviews, the researchers noticed that participants often described their behaviour when reflecting on the photographs. Within the behavioural patterns of participants, multiple self-management abilities and coping strategies were detected. These self-management abilities and coping strategies were in line with the definition of positive health to self-manage and adapt in the face of physical, social and mental challenges (Huber et al., 2011). We decided to use the Self-management of Wellbeing (SMW) theory of Steverink, Lindenberg and Slaets (2005) as a tool to code the self-management abilities of the participants within the transcripts of the interviews. SMW theory distinguishes six key self-management abilities: self-efficacy beliefs, a positive frame of mind, taking the initiative, investment behaviour, multifunctionality of resources, and variety in resources. Being self-efficacious refers to one’s belief in one’s competences and pursue of goals. Having a positive frame of mind refers to having a positive view or positive expectations towards the future. Taking the initiative refers to one’s behaviour to actually taking action, and is the opposite of being passive. Investment behaviour is the behaviour one undertakes to invest in resources for realizing certain aspects of well-being. Taking care of multifunctionality of resources refers to resources that serve both physical and social aspects of well-being simultaneously, such as visiting a sports club together with friends. Taking care of variety in resources refers to the ability to have more than one resource for realizing certain aspects of well-being, such as having both a partner and a very good friend to obtain affection from. All six key selfmanagement abilities were used to code the data.

Second, during participants’ descriptions of their behaviour, we also noticed the ability to cope with physical, social or mental challenges. Therefore, we determined whether the participant described active or passive strategies when adapting to situations, and coded these strategies as passive or active coping styles. In general, coping can be seen as the set of efforts people engage in to minimize the physical, psychological, or social harm of an event or situation (Lazarus & Folkman, 1984). In the context of this study, active coping strategies consisted of behaviour which was problem-solving, taking responsibility for the problem and trying to control one’s problems. Passive coping strategies consisted of avoidance behaviour or relying on other people to solve the situation.

After coding all the interviews, summaries of the codes were connected to predetermined themes and described in a scheme. The themes were: *Physical health, Social health, Mental health, Ability to adapt and self-manage, Awareness of health and Financial situation and health*. All members of the research team checked the scheme. In the next step of the analysis, codes were categorized to subthemes. Such as the subtheme “social contacts” for the theme Social Health. Using both the subthemes and themes, patterns were found and described as results. According to Malterud (2001), the positionality of the researcher (background and beliefs) may influence the analytical process and the research outcomes. The gap between the age of the interviewer and participant in the current study was quite large (mean of 52 years). Therefore, it might be that the interviewer did not understand the reflections and/or associations of the participants because they often referred to situations that happened in a historically different time. An example is for instance, a reflection about smoking habits from a 91 year old participant. When the participant was around twenty years of age, smoking was seen as normal at that time and had a different social status. The perception on smoking and health has changed throughout the years. In order to be aware of the effect of the researcher positionality on the data, the interviewer discussed the coding and participants reflections with members of the research team. In addition, any reflections about the positionality of the interviewers were discussed within the research team, to increase the transparency of the results (Holmes, 2020).

## 3 Results

### 3.1 Characteristics of the participants

All 19 participants were community dwelling and living in the northeast of the Netherlands. The mean age of the participants was 77 years (SD = 7.9), ranging from 61 to 90. Two participants requested their partner to be near during the interview to feel more comfortable. These interviews were, therefore, conducted with two participants at the same time. We compared the answers of participants who were interviewed together with those of the individual interviews, but we did not find any differences. Therefore, we counted the participants who were interviewed together as two individual interviews. All 19 participants were Dutch, although one participant was originally from Suriname and emigrated to the Netherlands when she was around the age of 20. The characteristics of the participants are presented in Table II.

**Table II.** Characteristics of the participants (n = 19)

Characteristics		*N*
Gender	Male	7
	Female	12
Age	60–70	3
	71–80	8
	81–90	8
Partner Status	Widow/widower	7
	Married and living with partner	11
	Found new love	1
Financial situation	Old age pension [Dutch: Algemene Ouderdoms Wet (AOW)]	6
	Did not gave full disclosure about financial situation	13
Educational level	Lower	11
	Middle	8
Previous job sector	Facility	2
	Industry	2
	Sales & retail	6
	Service	1
	Agriculture	1
	Education	2
	Nursing & care	5

### 3.2 Findings

Overall, all participants reflected on their perceptions of physical, social and mental health with the use of the photographs. First, the perceptions and experiences of the participants on these domains were described with the use of subthemes. Second, when analysing the data, patterns were found in self-management abilities and awareness on health. After the description of the perceptions, these patterns were described and illustrated with the use of quotes and examples.

## 4 Physical health

Twelve out of the 19 participants currently participated in sports activities such as gymnastics for older adults, tennis, exercising with a physiotherapist, and daily physical activities such as cycling and walking. For them the main goal of exercising was to keep the body’s strength, endurance and flexibility for as long as possible.

“*I participate in gymnastics to keep up with my physical health, I am 74 years old so I try to do what I still can”* (widow, 74 years old, P14).

Although the overall opinion was that exercising is healthy for all older adults, some participants (7 out of 19) also claimed that older adults should avoid getting injuries that might be more difficult to recover from at an older age.

*“You must be sure that you do not have any accidents on such an age. You have to take care of your health in a normal way”* (widower, 90 years old, P1).

Consequently, these participants did not dare to be involved in exercise or habitual physical activity. Most of the participants, who did exercise, enjoyed exercising in groups. Especially female participants mentioned that exercising in groups helped to relieve stress, to be able to talk about private problems with members of the group, be more competitive during exercising and to broaden social contacts, which contributed to both physical health and social health.

*“For me it is the good atmosphere. We are all older adults and every Wednesday we sit together, play tennis and afterwards drink coffee together” (*widow, 84 years old, P17).

### 4.1 Acceptance of physical disabilities

Many participants reported having physical disabilities. They claimed that at an older age, one must accept and deal with physical disabilities. For three participants, especially those who participated in sports at a professional level at a younger age; it was harder to accept declines in physical fitness. For instance, one participant (widower, 83 years old, P2) who was a professional football player in his early years claimed that he did not want to engage in sports anymore, and lost all interest in sports, except for watching sports on the television. Another participant claimed that sports was a “S*hip that had sailed”* (widower, 90 years old, P1), and said he thought that sports is inappropriate at his age. Furthermore, another participant explained that she has a very hard time accepting her physical limitations after working out in a gym and eating healthy all her life. She felt her body abandoned her after investing so much time in a healthy lifestyle.

*“It is very hard for me to accept and give it a place in my head that my body doesn’t function anymore. I felt so betrayed by my body. That feeling lasted for months* (female, married, 66 years old, P11).

### 4.2 Physical disease = bad luck

Almost all participants, 18 out of 19, said that disease accidently “happened” to them. They experience a lack of influence on their own health and disease in their personal life or that of their spouse. One male participant (87 years old, married, P3) claimed that a disease is just “*Bad luck*”, while another participant claimed, *“I do not have any control on my body; I feel different everyday” (*female, married, 72 years old, P8). These claims seem to be illustrations of a lack of awareness of personal abilities to adapt and self-manage physical challenges of

life.

## 5 Social health

### 5.1 Social contacts

Many participants talked about their family, friends, acquaintances and neighbours. Social contacts with their children and grandchildren meant a great deal to these participants in terms of feeling well.

*“It gives me great pleasure to do activities with my grandchildren. That makes me so happy”* (male, 81 years old, P6).

Besides, most participants stated that they are proud of their children and grandchildren.

*“You are even more proud on your grandchildren than on your children”* (widower, 90 years old, P1).

Some derived a kind of social status from the achievements of their children and grandchildren, for instance, having a child who is married to a partner with an important social position, or having a grandchild who graduated from university. For some participants, neighbours are important social contacts, contributing to feeling well.

*“I am glad that I live here, the people in the neighborhood are nice and always want to talk with you”* (widow, 84 years old, P17).

Some participants mentioned that their friends and acquaintances have been important contacts ever since their children grew up, and the bonds of those friendships have grown strong throughout the years.

*“These friendships have grown with us throughout our lives”* (female, 72 years old, married, P8).

There seemed to be a difference in the size of the social network of participants who kept living in the same house or area, and participants who moved to a new place. The size of the social network of participants who kept living in the same area seemed to be larger. Participants who moved to a new living environment mentioned that it was hard to get involved in new social contacts. Practical issues, such as remembering names of people, could play a role in attaining new contacts. *“It is difficult to recall names of new people*” (widow, 84 years old, P17).

### 5.2 Loneliness

Some participants explained that feelings of loneliness occurred when they were alone for a longer period. A number of participants noticed that they could not express their feelings (emotional loneliness). Especially when there was limited contact with nuclear and extended family members, or lacked visits from other people.

*“When you are alone for the entire weekend and nobody visits, you can feel lonely”* (widow, 72 years old, P5).

Some participants seemed to be aware of their own role in coping with loneliness. Especially for widowed participants, their social network and participation in (social) activities are important means to reduce loneliness.

“*I participate in gymnastics, playing cards and bingo. Just as I said, you have to stay involved in social activities”* (widow, 72 years old, P5).

Participants with a partner participated in (social) activities less frequently as widowed participants. Noticeably, participants with a partner did not talk about participating in activities to reduce loneliness.

## 6 Mental health

### 6.1 Family happiness and sorrow

When participants were asked about the impact of family on health, most participants mentioned that family could cause happiness, but also sorrow and worries.

*“Family brings you both joy and sorrow”* (widow, 61 years old, P14).

Several participants associated mental health with the worries that family can cause, such as work of children, education of grandchildren, or health of family members.

*“We had worries about our children when we were younger, for instance, about their clothes or their education. Now it is the same with the grandchildren”* (female, married, 72 years old, P8).

However, family was mentioned to be one of the main things, which made participants experience happiness.

*“With grandchildren, you get up from your chair and do activities together. You have a lot of fun with grandchildren, it makes me very happy”* (male, married, 81 years old, P6).

Four participants discussed their experiences with family feuds, and made clear how the feud affected their mental health. *“I had everything accepted for a while [emotions about the death of her partner], but now … .my sister made everything worse again”* (widow, 72 years old, P5).

### 6.2 Dealing with the loss of a loved one

Seven participants mentioned that they lost their partner. One of them found a new love. Participants who lost their partner reported to experience feelings of loneliness, decline of their social network, loss of energy, not knowing what to do in the future, and experiencing difficulties with accepting.

*“I wanted to stay in bed after the death of my husband and stay there”* (widow, 84 years old, P17).

The experience of mourning differed; it seemed that participants who undertook activities and searched for social contacts had a shorter period of mourning. For older adults who had a smaller social network it seemed to be more difficult to undertake activities, and for them the mourning period was longer and more intense. Most female participants mentioned that they proactively searched for informal or formal help to cope with their grief.

“*I go to a social worker to get help with the loss of my husband. I asked the general practitioner if I could go to someone who could help me. I am not going to wait until I feel completely down”* (widow, 73 years old, P12).

Male participants who lost a loved one seemed to be less aware of the mourning process and showed avoidant coping towards their loss.

“*The loss of my daughter, that was just something”* (male, married, 87 years old, P7). The death of their loved ones (especially the loss of the partner) had a big impact on the plans of the widowers.

“*When my wife still lived we had so many plans. She died suddenly. It shattered every plan we had”* (widower, 90 years old, P1). Most widowers showed no signs of proactively seeking informal or formal help. Support of children helped widowers in the mourning process. For one widower, a new love helped with the acceptance of loss.

“*I just sat there in the chair for all summer, doing nothing. I was not able to stand up anymore. My children said that I needed to do things again. Well I did, but nothing worked for me. I could go anywhere, but I was still alone. Then suddenly, I walked into my new girlfriend and everything changed”* (widower, 83 years old, P2).

## 7 Financial status and health perception

Twelve out of the 19 participants made clear that they needed to handle their finances carefully in order to make ends meet. Seven participants did not give full disclosure about their income. Some participants gave illustrations of their low income, such as having a tight budget for groceries, watching out for increasing prices at supermarkets, participating in free activities or saving on health care insurance. Moreover, some participants claimed that they needed to save money for expenses such as anniversaries, wheelchairs, healthcare and holidays. Although most of participants did have a tight budget, they all explained that money is not the most important issue in the world, and that they are satisfied with their income. In fact, some participants, especially males, stated that money “*does not interest them*”. None of the participants mentioned how different life could be if they would have more financial possibilities.

*“I do not care if I have thousand, ten thousand or fifteen thousand, you can spend what you have and nothing more”* (Male, married, 77 years old, P9).

### 7.1 Financial possibilities and health

Only two participants stated that money has no relationship with health what so ever. The other participants described associations with financial possibilities and better health care, health insurances, tools to make life easier, and healthy food. These participants explained that with more money you could buy “tools” such as wheelchairs or electric bicycles to make life easier. Especially, health insurances are associated with financial possibilities and health. These participants stated that a higher income gives better options for health insurance and health care facilities.

*“In these times, money is important for your health. Nowadays, you can buy your own health with use of your health insurance and how much coverage you pay for. If you cannot afford more than your basic health insurance, there are many things you cannot go to anymore” (*female, married, 72 years old, P8).

## 8 Ability to adapt and self-manage

All participants explained how they managed their health while giving descriptions of their behaviour with regard to physical, social and mental health. The self-management abilities of the participants are described along the six key self-management abilities of SMW theory of (Steverink, Lindenberg and Slaets, 2005): self-efficacy beliefs, a positive frame of mind, taking the initiative, investment behaviour, multifunctionality of resources and variety in resources.

Some participants described their belief in their own competences to pursue health- and well-being-related goals, which can be defined as the self-management ability being self-efficacious. These participants reported to belief in their own abilities to exercise, to maintain social contacts and to control their (negative) emotions. Almost all participants who described health-related goals showed signs of taking the initiative to pursue their goals.

*“I notice that I have to exercise to stay flexible, therefore I go to the exercise* g*roup”* (widow, 84 years old, P17).

Taking the initiative is a self-management ability, which 17 out of the 19 participants referred to. These participants explained how they take the initiative to stay healthy by going to sports clubs or participating in social activities.

*“I go to Bingo and we make cards in a group. Sometimes they invite a singer where I go to”* (widow, 74 years old, P4).

Most participants also talked about taking the initiative to spend time with family, partner, children and grandchildren.

*“We invite our children and grandchildren for dinner often”* (Male, married, 81 years old, P6).

By participating in long-term activities or investing in social relations, these participants also showed investment behaviour. Two participants do take initiative; however, these participants do not show investment behaviour.

*“I do want to help people; I help my neighbor when she needs me even in the middle of the night. They also asked me multiple times to become a member of the board, but I do not want any more”* (widower, 83 years old, P2).

Female participants showed more structural investment behaviour that resulted in a “safety net” when social contacts are declining. This was visible in how they invested in their social network through participating in (social) activities such as joining sports clubs, doing volunteer work, and playing games with inhabitants of the community.

“*It was always so fun to go to the rehearsals of the choir, it felt like family. During breaks, you could talk to everyone”* (Female, married, 83 years old, P14).

The male participants who lived with their partner were less often involved in social activities as the female participants. Most of the time, these male participants claimed that they did not have an interest in social activities or they did not like the obligations to participate in social activities.

*“I walk and cycle alone, I do not want to make appointments with other people, I am a free man”* (male, married, 87 years old, P3).

Noticeably, the social network of male participants who lost members of the network became small.

*“I have no friends anymore, just one friend who lives in a nursing home, but you can’t do anything with him. I am alone”* (widower, 83 years old, P2). Children and grandchildren were sometimes the only social contacts left.

The self-management abilities taking care of multifunctionality of resources and taking care of variety in resources were described by both male and female participants. Multifunctionality of resources, combining domains of well-being (social and/or physical well-being), became most apparent when participants mentioned being involved in sports groups or family activities.

*“I exercise for the mind and the contact with other people”* (female, married, 66 years old, P10).

Some participants had a limited variety in resources for some domains of well-being. Throughout the interviews, some participants showed for instance, difficulties to obtain more resources for affection when their social network became smaller.

*“If there are no social contacts any more, than that’s it”* (widower, 83 years old, P2). Notably, participants who showed the self-management abilities investment behaviour and taking the initiative, also showed more in resources for domains of well-being.

With regard to a positive frame of mind, only some females mentioned to have positive expectations towards the future, such as having faith that the future will be bright, or that sadness about the death of relatives will eventually pass off. *“I would like to live for twenty years or longer when I feel like this”* (widow, 74 years old, P4). These participants explained their positive frame of mind most when talking about coping with the loss of a close relative.

### 8.1 Ability to adapt

The other important positive health behaviour is “the ability to adapt” (Huber et al., 2011). One aspect of the ability to adapt is one’s strategy to solve problems. When dealing with (mental) health problems, participants showed both active and passive coping strategies. When asked what they did when it was needed to deal with (mental) health problems, nine participants talked about putting the situation into perspective, seeking help, searching for more information online, and talking with others.

*“I try to put things into perspective when dealing with problems*” (female, married, 66 years old, P11).

All these strategies can be classified as active coping strategies. However, ten participants also stated that they ruminate, think about revenge, ignore the situation, or avoid any confrontation when dealing with problems, or go out into nature to clear their minds.

*“I count to ten when I encounter problems or I go outside to cycle*” (male, married, 81 years old, P6).

In general, the male participants showed more characteristics of passive coping strategies than the female participants did, although four out of the seven male participants claimed to experience no worries or stress at the moment.

### 8.2 Awareness about control over health

To self-manage health-related situations, awareness of one’s own influence on health seems to be beneficial. The interviews revealed that most participants were not aware of the control they could have over their health. Participants were asked at the end of the interview whether they felt to have control over their physical, social or mental health. Only one participant stated that she has control over her mental and social health by actively participating in volunteer work as a board member of the elderly association and trying to put things into perspective when feeling down.

*“I do have control on my loneliness, I am a member of the board and go to the Christmas market with the board, I try meet up with people”* (female, married, 66 years old, P11).

Other participants stated that disease just happens, and you need to deal with the consequences that come along. Although health is something that can be controlled to some extent, these answers indicate that most participants were not aware of the influence they can have on their own health. Some participants even indicated that they have trouble understanding the question and the meaning of control over their health. Some participants claimed that they are in control over various or all aspects of their health, but could not explain how they do this. These answers seemed to be reflections of aspects of their lives they were happy with, rather than indications of how they controlled these aspects.

## 9 What is the perception of health?

When exploring the perceptions of health of the older adults with low SES in the current study, we noticed that there is not one unequivocal perception of health. For example, when discussing the photograph with the older adult in the wheelchair, the interviewer asked whether the older adult in the wheelchair could be considered healthy. Participants gave a variety of answers, such as “*No, he is not healthy because he is in a wheelchair”* (widow, 47 years old, P4). Alternatively *“Yes, it is not visible that he does not want to do anything anymore; he can still enjoy life”* (widow, 61 years old, P14). The answers varied between having good mental health, being able to do what you want, being physically healthy, having no cognitive impairments such as dementia, enjoying life, and being able to eat. Although participants gave a huge variety of answers on what the perception of health entailed, we did discover some commonalities among the perceptions of health.

First, the perception of health seemed to be dependent on the personal experience with diseases or the experience of members of the social network, such as having a relative who suffers from dementia. Besides the experience with health-related issues, cultural background also seemed to influence the perception of health. For example, the only participant who mentioned a relationship between the ability to eat and health originally came from Suriname.

Furthermore, the participants’ financial situation seemed to influence the perception of health. For instance, some participants mentioned that with more money one could afford better healthcare. Overall, it seemed that the perception of health was largely experience-oriented and depended on the background of the participant.

Second, during the reflections of participants, we noticed that most participants associated health with negative physical aspects, such as disease, physical impairments, but also with negative aspects of mental health, such as dementia, stroke or depression. Positive aspects of social and mental health, such as the joy family gave, were mentioned to be important for the wellbeing of the participant. Especially when the physical health of the participant declined, participant mentioned the importance of social contacts more often. However, participants did not explicitly made the connection between these positive aspects and health. Therefore, it seems that the perception of health is explicitly associated with the negative physical and mental aspects of health and no connections were made with the more positive mental and social aspects of health.

Third, most participants did not explicitly showed awareness of how their actions can influence their health. When asked whether they feel that they have any influence on their health, most participants explained that health “happens” to them. Instead of trying to influence their health, participants seemed to adapt to and accept health-related situations, such as their monthly budget, physical possibilities and their social network. Many participants showed signs of “cognitive dissonance reduction” (Festinger, 1957; McGrath, 2017), where contradictory beliefs, values or actions of participants are changed to become consistent and reduce psychological discomfort. For instance; claiming to have no interest in participating in sports anymore at an older age, likely due to physical limitations, while emphasizing the importance of engaging in physical activity in general. Because many participants were not aware of being able to influence certain challenges of life, they mostly accept their current situation, while remaining unaware of (possible) abilities to self-manage domains of health. Although most of the participants were not aware of their influence on health, all participants also showed self-management abilities (as distinguished by SMW theory). For instance, participants who stated to have no control over their social life did go to exercise groups to meet up with people to reduce loneliness. Therefore, it can be concluded that all participants self-managed aspects of physical, social and mental health in a kind of “automatic” way. The self-management of participants seemed to be driven by the health experiences of the participant or their social network. For instance, participants who mentioned to seek for mental help when dealing with the loss of a loved one because of the mourning experiences of their family members. We choose to use the term “automatic” because it seemed that participants did not make rational decisions to self-manage their health. Participants behaved more intuitive towards health-related situations without giving much thought about why and how they should manage their health.

## 10 Discussion

This study served as a step towards a better understanding of the health perceptions of older adults with a lower SES. The use of photographs provided the base to discuss aspects of physical, social and mental health. In total, 19 participants discussed their perceptions, experiences and beliefs about health during the interviews. Results suggested that the perception of health in older adults with a lower SES depended on the background of the participant, was predominantly experience-oriented and was mostly focused on the negative aspects and barriers of physical and mental health, such as physical limitations and depression. Participants mentioned social contacts as an important part of their well-being, but most participants made no connection between social contacts and health. Although the awareness of the participants’ influence on health was limited, all participants showed at least some automatic self-management ability to manage health-related situations.

In general, there seemed to be a large variety in perceptions of health, but we also discovered several commonalities among participants. First, perceptions of health depended on the personal background of the participants, such as cultural background and financial status. For instance, some participants mentioned that with more financial possibilities they could afford better healthcare. In addition, the health perceptions of participants were mostly experience-oriented, influenced by personal experiences with disease and health experiences of their social network members. Our findings are in line with the study of Flinterman et al. (2019), which also showed that the importance of positive health aspects of adults with low SES, such as the importance of physical and social health, varied between participants depending on cultural background, financial status and health status.

Second, most participants associated health with negative physical and mental health aspects, such as physical impairments, disease, dementia, depression and stroke. Although other research showed that older adults perceived health in a multidimensional way (Borawski et al., 1996), our finding that participants’ perceptions of health focused mainly on physical aspects is in line with results of the study of Huber et al. (2016). In which all stakeholder groups, such as health professionals and policymakers, considered “bodily functions” as the most important representation of positive health. Our findings are also in line with the study of Flinterman et al. (2019), in which adults with a lower SES explained the meaning of positive health mainly in terms of the medical representation of physical and cognitive health, such as “having no cognitive and physical disabilities”. Consequently, older adults with a lower SES perceive health primarily in terms of the biomedical model of health, which focusses on physical and biological aspects of health and the absence of disease (Wade & Halligan, 2004).

Third, although participants’ health perceptions focused on the negative aspects of physical and mental health, social aspects of health seemed to be most important for the participants’ well-being. Participants reported feelings of happiness interacting with children and grandchildren and referred to the importance of meeting with people to reduce loneliness. Furthermore, social contacts were frequently reported as one of the reasons to participate in group (physical) activities. The importance of social health for well-being in older adults is in line with the results of the study of Douma et al., 2017), in which social life was mentioned to be the most important domain of subjective well-being. Other studies also suggest the importance of social relations for quality of life at an older age (Wilhelmson et al., 2005) and the importance of social resources for successful ageing (Jopp et al., 2015).

Noticeably, the current study also showed that the relevance of social contacts increased when the physical health of participants declined. For instance, participants who suffered from physical impairments often referred to the importance of meeting with grandchildren and children, and the positive effects of these meetings on their mental state.

This finding is in line with a study of Huber et al. (2016), who found that spiritual and social aspects of positive health were more important for adults with a chronic disease than for adults without a chronic disease. In short, older adults with a lower SES did not explicitly connect their social contacts with health, but social contacts were an important contributor to their well-being, especially when physical health declined.

Fourth, most participants stated that they could not influence their health and that health is something that “happens” to them. Consequently, most participants accepted their health problems, and sometimes even changed their attitudes towards aspects of health to reduce cognitive dissonance. However, participants also showed various self-management abilities on physical, social and mental health aspects. For instance, a participant who invested time in her social network to reduce feelings of loneliness stated to have no control over her social health. In these situations, most participants showed intuitive behaviour when dealing with health-related situations rather than making rational decisions. This intuitive behaviour seemed to be influenced by previous personal health-related experiences or observed within their social network. In other words, it seemed that participants self-managed physical, social or mental health challenges automatically without being aware of doing so.

### 10.1 Strengths and limitations

Although the current study yielded important insights into the perceptions of positive health among older adults with a lower SES, it has some limitations as well. First, most participants were recruited at sports clubs or other activities for older adults, which likely has resulted in the recruitment of participants that are on average more active in, and aware of, maintaining their physical or social health. This may cause a selection bias, which potentially affected the results.

Second, although almost all participants explained that they enjoyed the PEI method, some participants were unsure about their responses during the interview. These participants asked for instance, whether they gave the right answers when reflecting on the photographs. *“Did I give the right answer? That you need to stay active”* (widower, 83 years old, P2). One participant indicated to have difficulties understanding the choice of the photographs by the researchers and the purpose of the study. For future research using PEIs or other creative methods with older adults with a lower SES, it could be considered too brief participants before the start of the interview instead of afterwards to avoid any misunderstandings about the procedure or purpose of the interview. However, we decided to brief participants after the interview, because our goal was to gain insights in the perceptions on several domains of health. By debriefing participants, we avoided mentioning the word health before the interview, and avoided directing participants’ thoughts in certain directions.

Third, it should be acknowledged that the choice of photographs affects the research process and outcomes (Church & Quilter, 2021; Padgett et al., 2013). With the use of preselected researcher-gathered photographs, we had the possibility to address various aspects of health. However, it is possible that we missed certain perceptions that participants might have had concerning health. Participant-generated photographs, also known as “photo voice*”*, is a different strategy within the PEI method (Church & Quilter, 2021; Evans-Agnew & Rosemberg, 2016), which give a “voice” to the involved participants. For future research, it could be informative to discover which photographs older adults with a lower SES would gather/produce when thinking about their meaning of health, and investigate whether this strategy would reveal different or additional perspectives of health.

Despite these limitations, several strengths of the current study should be mentioned. First, by the creative method of the PEI, we were able to learn more about the health perceptions of older adults with a lower SES. Using this method, we gained insights into the behaviour and experiences of the participants. Participants stated that they enjoyed the method, and some participants mentioned that reflecting on health was something they never did before. *“I have the feeling I take something out of this experience”* (male, married, 81 years old, P6). These positive experiences of participants seem to indicate that the use of photographs facilitated the interviews with older adults with low SES, and therefore the PEI method might be a more preferred way to interview this target group compared to more conventional verbal or written methods. This study might even have given a broader or better understanding of beliefs, experiences and perceptions of health and well-being compared to the use of predominantly verbal or written methods. However, this comparison cannot be confirmed as, to our knowledge, no other study used the PEI method in this target group. Another strength of this study is that the design of the PEI was developed and tested thoroughly before the interviews with participants. The photographs used in the study were selected with the help of six representatives of the target group, and a research team with five experienced researchers. The interview was tested with the target group two times prior to the start of the study. Furthermore, all members of the research team were closely involved in the analytical process, in order to increase the trustworthiness of the results (Amankwaa, 2016).

### 10.2 Recommendations for future research

Based on our experiences and methodological lessons learned, we recommend the following for future researchers using the photo-elicitation method with low SES older adults. First, it is recommended to work together with the target group throughout the whole research process, especially when selecting the photographs. This increases the chance that the visual tools suit the experiences, perceptions and reality of the target group. Second, we recommend to find a good balance between informing participants properly at the beginning of the interview, while avoiding framing participants’ associations before the start of the interview. We noticed that some participants kept asking questions during the interview about whether they gave the right answer. Therefore, it is recommended to test how and when to inform participants adequately about the study (before the study or debriefing afterwards).

### 10.3 Practical implications

There are many health-promotion interventions aiming to improve the health of older adults. However, the effectiveness of an intervention will depend on how it is tailored to the needs of the target group. Therefore, insight into the perceptions of older adults with a lower SES are important for health-promotion interventions to fit the needs and wishes of this target group (Milat et al., 2015). This study showed that the perceptions of health depend on the background of participants, is experience-oriented and is mainly focused on negative aspects of physical and mental health. Aspects of social health were mentioned to be most important for one’s well-being, although not directly linked to health. The PEI method seemed to be a proper facilitator for older adults with a lower SES to talk about experiences, health perceptions and self-management abilities. Therefore, this method can be used to investigate what health exactly entails for an older adult who wants to participate in a health-promotion intervention, in order to ensure that the intervention fits this person’s health perception.

With the shift from a welfare state to a participation society in the Netherlands, positive health is a topic high on the agenda of health care organizations and policymakers. Within a participation society, older adults are expected to live independently at home for as long as possible, and are responsible for managing their own health (Delsen, 2016). Participants showed various self-management abilities in the domains of physical, social and mental health as automatic behaviour while often not being aware of their self-management abilities. Noticeably, most participants stated that they were not able to influence their health. Consequently, participants often accepted their health problems, and sometimes even changed their attitudes towards health to reduce cognitive dissonance. Within a participation society, older adults having a more accepting way to cope with health problems and a restricted awareness of their self-management abilities might become a vulnerable group when their health decreases. The results of this study suggest that they might not ask for support and, consequently, might not receive the support they need to manage their health. Therefore, interventions are needed which raise awareness about self-managing positive health, probably with a more experience-oriented approach. For older adults who are aware of their needs to improve their self-management abilities for positive health, effective interventions are available, such as the “Self-Management-of-Well-being” interventions (Frieswijk et al., 2006; Kremers et al., 2006; Kuiper et al., 2019; Schuurmans, 2004).

## 11 Conclusion

In this research, we use the photo-elicitation method to gain more insight into the perceptions of positive health for older adults with a lower SES. The results of this study show that the use of PEI is a suitable method, positively received by the participants, to explore health perceptions, health experiences, and self-management abilities. We discovered several commonalities among the perceptions of health. The perceptions of health were dependent on the background of the participants, were mostly experience-oriented, and focused on the negative aspects of physical and mental health. Social contacts were an important part of participants’ well-being, especially when physical health declined. Noticeably, participants did not explicitly connected social contacts and health. Additionally, most participants stated that they could not influence their health and, consequently, often accepted their health problems. However, all participants showed at least some automatic ability in the realm of health-related situations. Health-promoting interventions with an experience-oriented approach are needed for older adults with a lower SES who need more support to improve or become aware of their self-management abilities.

## Data Availability

The data that support the findings of this study are available from the corresponding author, upon reasonable request.

## References

[cit0001] Amankwaa, L. (2016). Creating protocols for trustworthiness in qualitative research. *Journal of Cultural Diversity, 23*(3), 121-127.https://web.b.ebscohost.com/ehost/pdfviewer/pdfviewer?vid=0&sid=4fd37d3f-7571-4537-ab68-a28444f7153e%40pdc-v-sessmgr01 29694754

[cit0002] Barrett, A. E. (2003). Socioeconomic status and age identity: The role of dimensions of health in the subjective construction of age. *The Journals of Gerontology. Series B, Psychological Sciences and Social Sciences*, 58(2), 101–17. 10.1093/geronb/58.2.S101 12646599

[cit0003] Baum, S. K., & Boxley, R. L. (1983). Age identification in the elderly. *The Gerontologist*, 23(5), 532–537. 10.1093/geront/23.5.532

[cit0004] Borawski, E. A., Kinney, J. M., & Kahana, E. (1996). The meaning of older adults’ health appraisals: Congruence with health status and determinant of mortality. *The Journals of Gerontology. Series B, Psychological Sciences and Social Sciences*, 51(3), 157–170. 10.1093/geronb/51B.3.S157 8620363

[cit0005] Boyatzis, R. E. (1998). *Transforming qualitative information: Thematic analysis and code development*. California, United States of America: SAGE Publications, Inc.

[cit0006] Church, S., & Quilter, J. (2021). Consideration of methodological issues when using photo-elicitation in qualitative research. *Nurse Researcher*, 29(2), 25–32. Church_etal_NR_2021_Consideration_of_methodological_issues_when_using_photo_elicitation_in_qualitative_research.pdf (northampton.ac.uk). 33569938 10.7748/nr.2021.e1729

[cit0007] Collier, J., & Collier, M. (1986). *Visual anthropology*. University of New Mexico Press.

[cit0008] Copes, H., Tchoula, W., Brookman, F., & Ragland, J. (2018). Photo-elicitation interviews with vulnerable populations: Practical and ethical considerations. *Deviant Behavior*, 39(4), 475–494. 10.1080/01639625.2017.1407109

[cit0009] Delsen, L. W. M. (2016). Realisatie van de participatiesamenleving. Hervorming van de verzorgingsstaat in Nederland. *Belgisch Tijdschrift Voor Sociale Zekerheid*, 2015(4), 767–797. https://repository.ubn.ru.nl/bitstream/handle/2066/160932/160932.pdf?sequence=1&isAllowed=y.

[cit0010] Douma, L., Steverink, N., Hutter, I., & Meijering, L. (2017). Exploring subjective well-being in older age by using participant-generated word clouds. *The Gerontologist*, 57(2), 229–239. 10.1093/geront/gnv119. 26329319 PMC5434489

[cit0011] Engel, G. L. (1977). The need for a new medical model: A challenge for biomedicine. *Science*, 196(4286), 129–136. 10.1126/science.847460 847460

[cit0012] Evans-Agnew, R. A., & Rosemberg, M. A. S. (2016). Questioning photovoice research: Whose voice? *Qualitative Health Research*, 26(8), 1019–1030. 10.1177/1049732315624223 26786953

[cit0013] Festinger, L. (1957). *A theory of cognitive dissonance* (Vol. 2). Stanford university press.

[cit0014] Flinterman, F., Bisscheroux, P., Dijkema, P., den Hertog, F., de Jong, M., Vermeer, A., & Vosjan, M. (2019). Positieve Gezondheid en gezondheidspercepties van mensen met een lage SES. *Tijdschrift Voor Gezondheidswetenschappen*, 97(34), 96–105. 10.1007/s12508-019-0232-8

[cit0015] Frieswijk, N., Steverink, N., Buunk, B. P., & Slaets, J. P. (2006). The effectiveness of a bibliotherapy in increasing the self-management ability of slightly to moderately frail older people. *Patient Education and Counseling*, 61(2), 219–222. 10.1016/j.pec.2005.03.011 15939567

[cit0016] Ghaljaie, F., Naderifar, M., & Goli, H. (2017). Snowball sampling: A purposeful method of sampling in qualitative research. S*trides in Development of Medical Education, 14*(3), 1-4. 10.5812/SDME.67670.

[cit0017] Glaw, X., Inder, K., Kable, A., & Hazelton, M. (2017). Visual methodologies in qualitative research: Autophotography and photo elicitation applied to mental health research. *International Journal of Qualitative Methods*, 16(1), 1–8. 10.1177/1609406917748215

[cit0018] Gong, F., Casteneda, D., Zhang, X., Stock, L., Ayala, L., & Baron, S. (2012). Using the associative imagery technique in qualitative health research: The experiences of homecare workers and consumers. *Qualitative Health Research*, 22(10), 1414–1424. 10.1177/1049732312452935 22851495

[cit0019] Harper, D. (2002). Talking about pictures: A case for photo elicitation. *Visual Studies*, 17(1), 13–26. 10.1080/14725860220137345

[cit0020] Hennink, M. M., Kaiser, B. N., & Marconi, V. C. (2017). Code saturation versus meaning saturation: How many interviews are enough? *Qualitative Health Research*, 27(4), 591–608. 10.1177/1049732316665344 27670770 PMC9359070

[cit0021] Holmes, A. G. D. (2020). Researcher positionality–a consideration of its influence and place in qualitative research–a new researcher guide. *Shanlax International Journal of Education*, 8(4), 1–10. 10.34293/education.v8i2.1477

[cit0022] Huber, M., Knottnerus, J. A., Green, L., Van Der Horst, H., Jadad, A. R., Kromhout, D., & Schnabel, P. (2011). *How should we define health? BMJ, (343)*d4163, 1-3. doi:10.1136/bmj.d4163. 10.1136/bmj.d4163 21791490

[cit0023] Huber, M., van Vliet, M., Giezenberg, M., Winkens, B., Heerkens, Y., Dagnelie, P. C., & Knottnerus, J. A. (2016). Towards a ‘patient-centred’operationalisation of the new dynamic concept of health: A mixed methods study. *BMJ Open*, 6(1), e010091. 10.1136/bmjopen-2015-010091 PMC4716212 26758267

[cit0024] Jopp, D. S., Wozniak, D., Damarin, A. K., De Feo, M., Jung, S., & Jeswani, S. (2015). How could lay perspectives on successful aging complement scientific theory? Findings from a US and a German life-span sample. *The Gerontologist*, 55(1), 91–106. 10.1093/geront/gnu059 24958719 PMC5994883

[cit0025] Kaplan, G. A., Turrell, G., Lynch, J. W., Everson, S. A., Helkala, E. L., & Salonen, J. T. (2001). Childhood socioeconomic position and cognitive function in adulthood. *International Journal of Epidemiology*, 30(2), 256–263. 10.1093/ije/30.2.256 11369724

[cit0026] Knoops, K., & van den Brakel, M. (2010). Rijke mensen leven lang en gezond. *TSG*, 88(1), 17–24. 10.1007/BF03089530

[cit0027] Kremers, I. P., Steverink, N., Albersnagel, F. A., & Slaets, J. P. (2006). Improved selfmanagement ability and well-being in older women after a short group intervention. *Aging & Mental Health*, 10(5), 476–484. 10.1080/13607860600841206 16938683

[cit0028] Kuiper, D., Steverink, N., Stewart, R. E., Reijneveld, S. A., Sanderman, R., & Goedendorp, M. M. (2019). Pace and determinants of implementation of the self-management of wellbeing group intervention: A multilevel observational study. *BMC Health Services Research*, 19(1), 67. 10.1186/s12913-019-3891-x 30683092 PMC6346574

[cit0029] Lazarus, R. S., & Folkman, S. (1984). *Stress, appraisal, and coping*. Springer publishing company.

[cit0030] Loef, M., & Walach H. (2012). The combined effects of healthy lifestyle behaviors on all-cause mortality: A systematic review and meta-analysis. *Preventive Medicine*. 55(3), 163–170. 10.1016/j.ypmed.2012.06.017 22735042

[cit0031] Lyu, J., & Burr, J. A. (2016). Socioeconomic status across the life course and cognitive function among older adults: An examination of the latency, pathways, and accumulation hypotheses. *Journal of Aging and Health*, 28(1), 40–67. 10.1177/0898264315585504 26006338

[cit0032] Malterud, K. (2001). Qualitative research: Standards, challenges, and guidelines. *The Lancet*, 358(9280), 483–488. 10.1016/S0140-6736(01)05627-6 11513933

[cit0033] Mannay, D. (2013). Who put that on there … why why why? Powergames and participatory techniques of visual data production. *Visual Studies*, 28(2), 136–146. 10.1080/1472586X.2013.801635

[cit0034] McGrath, A. (2017). Dealing with dissonance: A review of cognitive dissonance reduction. *Social and Personality Psychology Compass*, 11(12), 1–17. 10.1111/spc3.12362

[cit0035] Milat, A. J., Bauman, A., & Redman, S. (2015). Narrative review of models and success factors for scaling up public health interventions. *Implementation Science*, 10(1), 1–11. 10.1186/s13012-015-0301-6 26264351 PMC4533941

[cit0036] Padgett, D. K., Smith, B. T., Derejko, K. S., Henwood, B. F., & Tiderington, E. (2013). A picture is worth …? Photo elicitation interviewing with formerly homeless adults. *Qualitative Health Research*, 23(11), 1435–1444. 10.1177/1049732313507752 24122520 PMC3992880

[cit0037] Pain, H. (2012). A literature review to evaluate the choice and use of visual methods. *International Journal of Qualitative Methods*, 11(4), 303–319. 10.1177/160940691201100401

[cit0038] Präg, P., Mills, M. C., & Wittek, R. (2016). Subjective socioeconomic status and health in cross-national comparison. *Social Science & Medicine*, 149, 84–92. 10.1016/j.socscimed.2015.11.044 26708244

[cit0039] Schuurmans, H. (2004). Promoting well-being in frail elderly people: Theory and intervention. Universal Press. Retrieved September10, 2020, from http://dissertations.ub.rug.nl/faculties/medicine/2004/j.e.h.m.schuurmans/15704605

[cit0040] Steverink, N., Lindenberg, S., & Slaets, J. P. (2005). How to understand and improve older people’s self-management of wellbeing. *European Journal of Ageing*,2(4), 235–244. 28794738 10.1007/s10433-005-0012-y PMC5546286

[cit0041] Steverink, N., Westerhof, G. J., Bode, C., & Dittmann-Kohli, F. (2001). The personal experience of aging, individual resources, and subjective well-being. *The Journals of Gerontology. Series B, Psychological Sciences and Social Sciences*, 56(6), 364–373. 10.1093/geronb/56.6.P364 11682590

[cit0042] Swain, J. (2018). *A hybrid approach to thematic analysis in qualitative research: Using a practical example*. SAGE Publications Ltd.

[cit0043] Szanton, S. L., Seplaki, C. L., Thorpe, R. J., Allen, J. K., & Fried, L. P. (2010). Socioeconomic status is associated with frailty: The women’s health and aging studies. *Journal of Epidemiology and Community Health*, 64(1), 63–67. 10.1136/jech.2008.078428 19692719 PMC2856660

[cit0044] Statistics Netherlands (CBS) (2016). *Standaard Onderwijsindeling 2016*, 64(1), 63–67. https://www.cbs.nl/nl-nl/onze-diensten/methoden/classificaties/onderwijs-en-beroepen/standaard-onderwijsindeling--soi--/standaard-onderwijsindeling-2016

[cit0045] Terry, G., Hayfield, N., Clarke, V., & Braun, V. (2017). *The SAGE handbook of qualitative research in psychology: Thematic analysis. 5*(2), 17-37. London, England: SAGE Publications ltd. https://books.google.nl/books?hl=nl&lr=&id=AAniDgAAQBAJ&oi=fnd&pg=PA17&dq=Terry,+G.,+Hayfield,+N.,+Clarke,+V.,+%26+Braun,+V.+(2017).+The+SAGE+handbook+of+qualitative+research+in+psychology:+Thematic+analysis.+(2),+17-37.+London,+England:+SAGE+Publications+ltd.&ots=doi8qmziJ0&sig=Tz48S1LujTvupkd7avvkeDoQJdo&redir_esc=y#v=onepage&q&f=false.

[cit0046] Van Lenthe, F. J., Schrijvers, C. T., Droomers, M., Joung, I. M., Louwman, M. J., & Mackenbach, J. P. (2004). Investigating explanations of socio-economic inequalities in health: The Dutch GLOBE study. *The European Journal of Public Health*, 14(1), 63–70. 10.1093/eurpub/14.1.63 15080394

[cit0047] Von Faber, M., Bootsma–van der Wiel, A., van Exel, E., Gussekloo, J., Lagaay, A. M., van Dongen, E., & Westendorp, R. G. (2001). Successful aging in the oldest old: Who can be characterized as successfully aged? *Archives of Internal Medicine*, 161(22), 2694–2700. 10.1001/archinte.161.22.2694 11732934

[cit0048] Wade, D. T., & Halligan, P. W. (2004). Do biomedical models of illness make for good healthcare systems? *Bmj*, 329(7479), 1398–1401. 10.1136/bmj.329.7479.1398 15591570 PMC535463

[cit0049] Wardle, J., & Steptoe, A. (2003). Socioeconomic differences in attitudes and beliefs about healthy lifestyles. *Journal of Epidemiology and Community Health*, 57(6), 440–443. 10.1136/jech.57.6.440 12775791 PMC1732468

[cit0050] Wilhelmson, K., Andersson, C., Waern, M., & Allebeck, P. (2005). Elderly people’s perspectives on quality of life. *Ageing and Society*, 25(4), 585–600. 10.1017/S0144686X05003454

[cit0051] World Health Organization (WHO). 2006. Constitution of the World Health Organization. http://www.who.int/governance/eb/who_constitution_en.pdf

[cit0052] World Health Organization (WHO). 2018. World Health Organization: Ageing and health. https://www.who.int/news-room/fact-sheets/detail/ageing-and-health

[cit0053] World Health Organization (WHO). 2020. World Health Organization: Health 2020: A European policy framework supporting action across government and society for health and well-being. https://www.euro.who.int/__data/assets/pdf_file/0006/199536/Health2020-Short.pdf

